# Stakeholders’ perspectives on access-to-medicines policy and research priorities in Latin America and the Caribbean: face-to-face and web-based interviews

**DOI:** 10.1186/1478-4505-12-31

**Published:** 2014-06-25

**Authors:** Thiago Botelho Azeredo, Vera Lucia Luiza, Maria Auxiliadora Oliveira, Isabel Cristina Martins Emmerick, Maryam Bigdeli

**Affiliations:** 1 Pharmacy School, Federal University of Rio de Janeiro, 373, Av. Carlos Chagas Filho, Centro de Ciências da Saúde, Bloco K #c050, Cidade Universitária, 21941-902 Rio de Janeiro, RJ, Brazil; 2 Nucleus for Pharmaceutical Policies, National School of Public Health, Oswaldo Cruz Foundation, 1480, Rua Leopoldo Bulhões # 624, Manguinhos, 21021-000 Rio de Janeiro, RJ, Brazil; 3 Department of Population Medicine, Drug Policy Research Group Harvard Medical School, Harvard University, 133 Brookline Ave, 6th Floor, Boston, MA 02215, USA; 4 Alliance for Health Policy and Systems Research, World Health Organization, 20 avenue Appia, 1211 Geneva, Switzerland

**Keywords:** Access to medicines, Health systems, Health systems research, Interviews, Latin America and the Caribbean, Priority setting, Web survey

## Abstract

**Background:**

This study aims to rank policy concerns and policy-related research issues in order to identify policy and research gaps on access to medicines (ATM) in low- and middle-income countries in Latin America and the Caribbean (LAC), as perceived by policy makers, researchers, NGO and international organization representatives, as part of a global prioritization exercise.

**Methods:**

Data collection, conducted between January and May 2011, involved face-to-face interviews in El Salvador, Colombia, Dominican Republic, and Suriname, and an e-mail survey with key-stakeholders. Respondents were asked to choose the five most relevant criteria for research prioritization and to score policy/research items according to the degree to which they represented current policies, desired policies, current research topics, and/or desired research topics. Mean scores and summary rankings were obtained. Linear regressions were performed to contrast rankings concerning current and desired policies (policy gaps), and current and desired research (research gaps).

**Results:**

Relevance, feasibility, and research utilization were the top ranked criteria for prioritizing research. Technical capacity, research and development for new drugs, and responsiveness, were the main policy gaps. Quality assurance, staff technical capacity, price regulation, out-of-pocket payments, and cost containment policies, were the main research gaps. There was high level of coherence between current and desired policies: coefficients of determination (R^2^) varied from 0.46 (*Health system structure*; *r* = 0.68, *P* <0.01) to 0.86 (*Sustainable financing*; *r* = 0.93, *P* <0.01). There was also high coherence between current and desired research on *Rational selection and use of medicines* (*r* = 0.71, *P* <0.05, R^2^ = 0.51), *Pricing*/*affordability* (*r* = 0.82, *P* <0.01, R^2^ = 0.67), and *Sustainable financing* (*r* = 0.76, *P* <0.01, R^2^ = 0.58). Coherence was less for *Health system structure* (*r* = 0.61, *P* <0.01, R^2^ = 0.38).

**Conclusions:**

This study combines metrics approaches, contributing to priority setting methodology development, with country and regional level stakeholder participation. Stakeholders received feedback with the results, and we hope to have contributed to the discussion and implementation of ATM research and policy priorities in LAC.

## Background

The achievement of the health-related Millennium Development Goals [1] strongly depends on health system capacity. Research for health has been recognized as crucial to generate innovations driven by health problems and diseases that disproportionally affect low- and middle-income countries (LMICs), as well as to provide the scientific and technical evidence needed to promote decision-making effectiveness regarding resource allocation, policies, and health system strengthening interventions [2-6].

Since 1990, the Commission on Health Research for Development identified a huge inequity in health research financing, with only 4.4% of global resources directed to health problems of developing countries. By 2000, this persistent imbalance between spending and disease burden was characterized as the ‘10/90 gap’ [7,8]. International political declarations call for increased and more equitable funding for research, and to make it more responsive to health systems needs in LMICs [3,4,7,9]. In this sense, setting research priorities at national, regional, and global levels is a core strategy aimed at closing the gap [5,7,10].

The delivery of medicines to people in need is an essential component to strengthen health systems effectiveness. Ensuring access to medicines (ATM) depends on efforts related to different domains such as sustainable financing, the existence of a network of reliable health services, and an efficient supply chain management [11,12]. Therefore, it is crucial to generate reliable and accurate information on different ATM domains that could be used for strengthening health systems. Yet, in order to make research responsive to health systems needs in LMICs, priority-setting processes can be useful for targeting research that presents the greatest potential public health benefit [10].

As part of a global exercise on priority setting for health policy and research on ATM in LMICs, coordinated by the Alliance for Health Policy and Systems Research (AHPSR) [13], two studies involving Latin American and the Caribbean (LAC) LMICs were carried out by researchers from the Sergio Arouca National School of Public Health, Brazil. One of them, published in 2013 [14], consisted of a scoping study that identified ATM research issues and gaps through the analysis of the scientific publication trends in LAC. It showed that, even though a lot of effort has been made and achievements have been reached in some countries, research on ATM still remains a challenge for countries in Latin America and the Caribbean. The second study, presented in this paper, aimed at ranking policy/research items, in order to identify policy and research gaps on ATM in LMICs of LAC, as perceived by policy makers, researchers, and non-governmental organization (NGO) and international organization representatives; the results of both were used in the global exercise [13].

The main objectives of the AHPSR global study were to identify priority policy issues in ATM relevant for LMICs, to identify research questions that would help address these policy issues, and to prioritize these research questions in a health policy and systems research (HPSR) agenda [13]. Different researchers have studied LMICs from other geographic regions.

## Methods

The data collection strategy conducted from January to May 2011 involved two approaches: face-to-face interviews with key-stakeholders from selected countries and an e-mail survey. Both approaches used the same questionnaire, in which a single list of items was ranked. Respondents were asked to score each policy/research item according to the degree to which it represented a current policy, a desired policy, a current research topic, and/or a desired research topic.

The methodology adopted in this study was adapted from Urcullo et al. [15], whose method was applied in a similar study on priority setting for HPSR, on health financing and human resources for health.

### Research settings and participants selection

Face-to-face interviews were carried out in four countries: El Salvador; Colombia, Dominican Republic, and Suriname. Country selection criteria covered the inclusion of both lower- (El Salvador) and upper-middle income countries (Colombia, Dominican Republic, and Suriname) from all Pan American Health Organization (PAHO) sub-regions (Central America, South America, and the Caribbean); existence of local researchers working on ATM and related issues; and agreement from country representatives to participate in the study. As medicines were seen as a sensitive political subject, PAHO officers mediated the identification of countries and the invitation to join the study.

In each country, interviews with 12 stakeholders were planned: five government decision makers (two high level decision makers at the Ministry of Health (MoH); one person in charge of pharmaceuticals at the MoH; one member of the national congress health commission; one person from the drug regulatory authority); one member of medical and one member of pharmaceutical professional associations; one representative of international organizations (World Health Organization/PAHO; USAID; Inter-American Development Bank; World Bank); two researchers working on ATM; and two NGO representatives. Local partners from PAHO offices supported the identification of interviewers.

The e-mail survey was carried out to expand the coverage of countries and key stakeholders. It was planned to include one person in charge of pharmaceuticals in the MoH and two activists from NGOs with relevant actions on ATM from each LMIC in the region (excluding the visited ones); experts with at least three publications on ATM in LAC in the previous 10 years; 23 international agency representatives (PAHO focal points on medicines); and persons from the pharmaceutical industry with ATM projects in the region. We were able to identify 112 potential respondents. After sending the first invitation, two follow-up contacts were made requesting a reply.

### Questionnaire structure

The first step in the development of the questionnaire was the selection of a list of items that would be subjected to ranking. Items were generated as exhaustively as possible and were phrased as to reflect both policy issues and research topics, pertinent to the discussion of ATM. Those policy/research items were developed in an AHPSR expert meeting, held in Cambodia in 2010 – a preparatory meeting for regional prioritization exercises, which were intended to subsidize a global prioritization effort on ATM research [13]. Regional team leaders agreed upon an analytical framework based on the WHO four ATM dimensions (rational selection and use, affordable prices, sustainable financing, and reliable health and supply systems) and determinants rooted in local, national, and international contexts [12]. For this regional exercise, items generated were organized in a matrix reflecting the analytical framework (Table 1).

**Table 1:** Policy/research items^* ^generated by the regional meeting in Cambodia according to the World Health Organization framework for access to medicines and Health System Level^**^

	**WHO Framework for ATM**			
	**1. Rational selection and use of medicines**	**2. Pricing/affordability**	**3. Reliable health system/health system structure**	**4. Sustainable financing**
**I. Individual, household, and community level**	1a) Information asymmetry (user knowledge level; communication and language barriers – as for ethnic minorities)	2a) High out-of-pocket payment	3a) Geographical accessibility: physical barriers; distance to facilities; remoteness (combination of physical barriers and infrastructure weakness)	4a) Community financing arrangements
		2c) Community participation in medicine delivery arrangements		
				4b) Role of government subsidies at community level
	1b) Health seeking behavior, preference for private care, preference for secondary level of care (bypass primary health care), self-medication			
				4c) Focal models of subside
			3e) Traditional medicine (e.g., unclear distinction between allopathy and non-allopathy)	
	1c) Beliefs about illness and treatment (traditional practices; demand for injections and branded medicines; prescribers and dispensers perception of quality of drugs)			
**II. Public and private health service delivery channels**	1d) Impact of advertisements on medicines; incentives (or lack of, leading to private or dual practice); medicines becoming a source in financing for health services	2d) Medicine price variation according to geographical location (e.g., urban/rural differences, higher prices in remote areas); price differential between public and private	3b) Distribution systems and supply chains	4d) Health provider payment methods
				4e) Health insurance coverage and models
			3c) Pharmaceutical services at local level	
	1e) Staff capacity for rational prescription and use of medicines; training curriculum		3d) Informal markets – substandard quality and counterfeit medicines	
			3f) Staff and technical capacity: for supply management; with managerial, interpersonal, and information technology (IT) skills; with local language skills	
	1f) Clinical treatment guidelines, and essential medicines list: development; incentives for implementation; operational mechanisms; standardization (including between private and public sectors)			
			3g) Responsiveness to patient needs; differential responsiveness between public and private	
	1g) Incentives for rational use of medicines and for implementation of generic policy			
			3j) Availability of medicines (especially in public sector)	
**III. Health sector**	1h) Staff deployment	2b) Opportunity costs	3h) Public-private mix: reliance on private sector delivery; public-private partnerships and their role in access to medicines	4f) Government budgets for medicines
		2e) Medicine price information system		4g) Reimbursement policies
	1i) Pharmacovigilance, information on adverse drug reaction, and other problems related to medicines	2f) Impact of prices on access		
				4h) Cost containment policies
			3i) Central procurement policies vs. decentralization	
		2g) Policy and regulation for medicines price		
			3k) Quality assurance	
		2h) Incentives for implementation of generic policy		
			3l) Coordination between health policies and medicines policies; referral policy/referral system	
	1j) Medicine information system (not only price information)/competing with medicines advertisement			
			3m) Monitoring and evaluation systems; funding for monitoring and evaluation	
**IV. Beyond the health sector**	1k) Intersectoral initiatives (e.g., rational use of medicines in schools – role of Ministry of Education)	2i) Patents and intellectual property issues	3n) Governance and governing: law and regulation enforcement; transparency and accountability	4i) Donor funding; harmonization and alignment; verticalized donor support
		2j) Finance policies: taxes, autonomy, privatization; exemption systems		
				4j) Patents and intellectual property issues
			3o) Regional integration and economic cooperation (example: UNASUL, MERCOSUL)	
				4k) Finance policies: taxes, autonomy, privatization
		2k) Trade and economic goals (impact of health sector policies outside the health sector)		
				4l) Trade and economic goals (impact of health sector policies outside the health sector)
			3p) Promotion of research and development – new drugs/neglected diseases.	
		2l) Budget allocation to health		4m) Budget allocation to health
		2m) Medicine production	3q) Medicine production	

*Item codes reflect the order in which they appeared in the questionnaire. Here they are presented according to Health System Level.

^**^Health System Levels according to Bigdeli et al. [12]: I. Individual, household and community; II. Health service delivery; III. Health Sector; IV. Beyond the health sector (Public policies cutting across sectors; and, International and regional level).

The questionnaire was composed of five parts: I) interviewee profile (background, educational level and affiliation); II) an open-ended question regarding access to medicines; III) ranking of research prioritization criteria; IV) policy priority rankings; and, V) research priority rankings. This paper discusses parts III, IV and V.

In part III, interviewees and respondents were provided with a list of prioritization criteria for research and asked to rank them, in an attempt to identify what should drive research priority definitions on ATM. It also elucidates which criteria were more likely to guide respondents’ rankings of the research alternatives.

In part IV, interviewees and respondents were provided with a list of items (Table 1) and asked to rank them, as they would represent policy priorities regarding the following aspects:

• Policies being carried out by the government (current policies);

• Policies that the government should be carrying out (desired policies).

In part V, interviewees and respondents were asked to rank the same list of items, but in this case, as they would reflect research priorities on ATM:

• Topics being investigated at national research centers (current research);

• Research topics required for public health policies (desired research).

In all ranking parts of the questionnaire, items could be added (by means of an “other” option) if participants felt that the provided list was incomplete.

All research materials were produced in Portuguese and translated into English and Spanish. For face-to-face interviews, a printed list of items was given to the interviewees in order to help them think about each item. Trained members of the research team, who performed pre-tests and meetings to standardize questionnaire application, conducted the interviews. The e-mail survey version contained explanations and instructions to assure a common comprehension and filling of the questionnaire.

### Ranking and scoring procedures

Interviewees and e-mail survey respondents were asked to choose the five most relevant criteria for research prioritization (part III) and policy/research items (parts IV and V), and sort them from 1 (top priority) to 5 (5^th^ priority). Selected criteria and policy/research items (Table 1) received a score for each interviewee/respondent (from 5 to 1, with higher scores for the most important and lower scores for the least important within the chosen list). Criteria and policy/research items that were not among the five most relevant received a score of 0.

We calculated average scores for each country visited as well as for the e-mail survey responses. Total scores for each criterion and summary rankings for each assessed aspect (current and desired policies; current and desired research) were obtained by summing those average scores.

### Coherence analysis

A coherence analysis was performed in order to answer the following questions:

1. How well does the government respond to the needs of the population? – By comparing answers about policies being carried out by the government (current policies) vs. policies that the government should be carrying out (desired policies).

2. Is scientific evidence being generated to support policies that respond to the needs of the population? – By comparing answers about policies that the government should be carrying out (desired policies) vs. topics being investigated at national research centers (current research).

3. Is research moving in the right direction? – By comparing answers about topics being investigated at national research centers (current research) vs. research topics required for public health policies (desired research).

Linear regressions depicted on scatter plots were performed to test these relationships. Strength and direction of association were estimated by calculating the correlation coefficient (Pearson’s *r*) and its statistical significance (*P* value). Data linearity fit was expressed by coefficients of determination (R^2^), used as coherence indicators.

We assumed that interviewees and respondents are legitimate spokespersons to indicate priorities on behalf of the population, and are informed enough to point out policies governments are currently carrying out and topics currently being investigated at national research centers.

The National School of Public Health Ethics Research Committee approved the project. All interviews were conducted after the signing of a written informed consent. In the case of the e-mail survey, the informed consent was presented to participants as the first page of the form and they were asked to fill it in if they agreed to participate.

## Results

Forty-nine interviews were completed in country visits (Table 2). Two of the selected stakeholders were unavailable in Suriname and El Salvador. In Colombia, a higher number of stakeholders were included, due to an increased interest and participation of NGO representatives during data collection.

**Table 2:** Number of respondents by country

**Information source**	**Number of respondents**
**Country visits**	**49**
Colombia	15
Dominican Republic	12
El Salvador	11
Suriname	11
**E-mail survey**	**35**
Argentina	3
Bolivia	1
Brazil	13
Colombia	3
Costa Rica	1
Ecuador	6
USA	4
Guatemala	1
Mexico	1
Peru	2
**Total**	**84**

Source: Authors.

From the target of 112 stakeholders, 98 e-mails were successfully sent (14 emails bounced), and a final response rate of approximately 35.7% was reached after at least two attempts. The final sample of respondents in the e-mail survey was mainly composed of researchers (48.6%) and NGO representatives (22.9%) – which expanded the representativeness of those types of stakeholders in the study, followed by international agency representatives (mainly PAHO officers; 14.3%), and MoH representatives (11.4%). There was a low representativeness of pharmaceutical industry respondents, with only one response.

### Research prioritization criteria

The highest ranked criterion that should be applied in the definition of research priorities on ATM was *Relevance*, which was also the most cited in Colombia, Dominican Republic, and in the e-mail survey (Table 3). The second criterion was *Feasibility*, and the third *Research utilization*. The latter, was also one of the top three ranked criteria in Colombia, Suriname, and in the e-mail survey. Whereas the first item implies a relationship between the measurement or magnitude of a problem and its degree of priority, the others refer to more operational or pragmatic aspects of research – denoting expectations towards the usefulness of policy research as an input to public management and decision-making.

**Table 3:** Respondent rankings of criteria that should be applied to define research priorities on access to medicines^*^

	**Items (proposed criteria)**	**Colombia**	**Dominican Republic**	**El Salvador**	**Suriname**	**E-mail survey**	**Total**
g	Relevance (magnitude of the problem; persistence of the problem; impact on health; urgency)	**1.5**	**1.8**	0.6	**0.9**	**2.0**	**6.7**
b	Feasibility (capacity of the system to carry out the research; funding support; justification of the cost/investment; justification of time)	**0.8**	1.0	0.8	0.7	0.7	**4.1**
i	Research utilization (adequacy and usefulness of the current knowledge base (avoiding duplication); applicability of the research outcome; availability of cost-effective interventions (access); operational effectiveness)	**1.0**	0.3	0.5	**1.1**	**1.0**	**3.9**
f	Political will/acceptability/commitment	0.5	**1.1**	**0.9**	0.8	0.5	3.8
c	Human rights issues; equity focus; ethical and moral issues	0.7	**1.3**	**0.9**	0.3	0.6	3.7
h	Responsiveness to the National Health Policy or national goals	0.7	0.8	0.4	0.8	**1.0**	3.6
a	Community concern/demand; environmental health and sociopolitical effects	0.3	0.3	**1.1**	**0.9**	0.4	3.0
d	Impact on development; economic impact	0.2	0.6	0.5	0.4	0.3	1.9
e	Partnership building; research capacity building	0.3	0.2	0.0	0.1	0.4	1.0

*Item codes reflect the order in which they appeared in the questionnaire. Here they are presented according to total ranking scores. Bold numbers represent the three highest scores in each setting.

Source: Authors.

In El Salvador, political and rights-based criteria were considered more important than managerial ones. These criteria were also high ranked in the Dominican Republic and in Suriname. Responsiveness to the National Health Policy was considered important in the e-mail survey (Table 3).

It is important to notice that criteria were identified and ranked for scoring policy relevance of research items were used implicitly by participants, and were scored separately (i.e., participants gave one overall score to each item according to the degree to which it was desired research, and not according to each criterion).

### Policy and research rankings

Table 4 shows the three top ranked policy/research items in each assessed aspect, as well as their summary rankings.

**Table 4:** Summary ranking scores for current policies, desired policies, current research, and desired research

Item codes^*^	***WHO Framework domain***	**Current policies**	**Desired policies**	**Current research**	**Desired research**
	**Items (policy or research topics)**				
	***Rational Selection and Use of Medicines***				
1f	Clinical treatment guidelines, and essential medicines list: development; incentives for implementation; operational mechanisms; standardization (including between private and public sectors)	**17.7**	**12.4**	**9.8**	**9.8**
1i	Pharmacovigilance, information on adverse drug reaction, and other problems related to medicines	**10.1**	**8.0**	**6.9**	**7.6**
1e	Staff capacity for rational prescription and use of medicines; training curriculum	**7.1**	**11.5**	3.0	**9.3**
1d	Impact of advertisements on medicines; incentives (or lack of, leading to private or dual practice); medicines becoming a source in financing for health services	3.3	7.3	**5.1**	7.0
	***Pricing/Affordability***				
2g	Policy and regulation for medicines price	**11.4**	**10.9**	**5.7**	**9.4**
2f	Impact of prices on access	**8.2**	**8.0**	**8.3**	**11.0**
2a	High out-of-pocket payment	7.6	**9.1**	5.3	**8.8**
2l	Budget allocation to health	**9.4**	6.0	4.6	4.3
2i	Patents and intellectual property issues	6.4	3.9	**7.1**	5.6
	***Reliable Health System/Health System Structure***				
3j	Availability of medicines (especially in public sector)	**8.8**	6.3	**8.3**	**7.2**
3a	Geographical accessibility: physical barriers; distance to facilities; remoteness (combination of physical barriers and infrastructure weakness)	**10.2**	**8.8**	3.9	**6.6**
3k	Quality assurance	6.8	**6.8**	**4.0**	**8.6**
3b	Distribution systems and supply chains	**8.5**	**7.9**	3.9	5.3
3f	Staff and technical capacity: for supply management; with managerial, interpersonal and information technology (IT) skills, with local language skills (English)	5.1	**6.8**	1.8	5.3
3c	Pharmaceutical services at local level	4.7	4.3	**4.0**	2.4
	***Sustainable Financing***				
4m	Budget allocation to health	**10.7**	**10.7**	**7.2**	**8.7**
4e	Health insurance coverage and models	**9.4**	**10.7**	**7.4**	**8.9**
4f	Government budgets for medicines	**10.8**	**11.0**	**5.4**	7.8
4h	Cost containment policies	6.9	9.1	5.1	**10.4**

*Item codes reflect the order in which they appear in the questionnaire. Top three summary scores in each domain marked in bold numbers.

Source: Authors.

One single item from the domain of *Rational Selection and Use of Medicines* scored the highest on almost all categories (current and desired policies, and current and desired research): (1f) Clinical treatment guidelines, and Essential Medicines List.

On the other domains, the highest ranked items regarding current policies were also the highest ranked for desired policies: (2g) Policy and regulation for medicines price – *Pricing*/*Affordability*; (3a) Geographical accessibility – *Reliable Health System*/*Health System Structure*; and (4f) Government budgets for medicines – *Sustainable Financing*.

With respect to current research, the main items were (1f) clinical treatment guidelines and essential medicines list; (2f) impact of prices on access; (3j) availability of medicines; and (4e) health insurance coverage and models.

In the domain of *Rational Selection and Use of Medicines*, the main desired research items were (1f) clinical treatment guidelines and essential medicines list, (1e) staff capacity and training, and (1i) pharmacovigilance, demonstrating an overall demand for investigations that focus on health service delivery and health sector levels. Regarding *Pricing*/*Affordability*, (2f) impact of prices on access, (2g) policy and regulation for medicines price, and (2a) high out-of-pocket payment received the highest summary rankings. In the domain of *Reliable Health System*/*Health System Structure*, the main topics required for the design of public health policies were first (3k) quality assurance and, second, (3j) availability of medicines; the third highest scored topic was (3a) geographical accessibility. When it comes to *Sustainable Financing*, stakeholders wanted to increase knowledge mainly on (4h) cost containment policies, (4e) health insurance coverage and models, and (4m) budget allocation to health. This demonstrates the interest in research focused on meso- and macro-level issues involved in *Sustainable Financing*, rather than micro-, health service, or community level topics in this domain.

### Coherence analysis

#### How well do governments respond to the ATM needs of populations?

**Figure 1:**
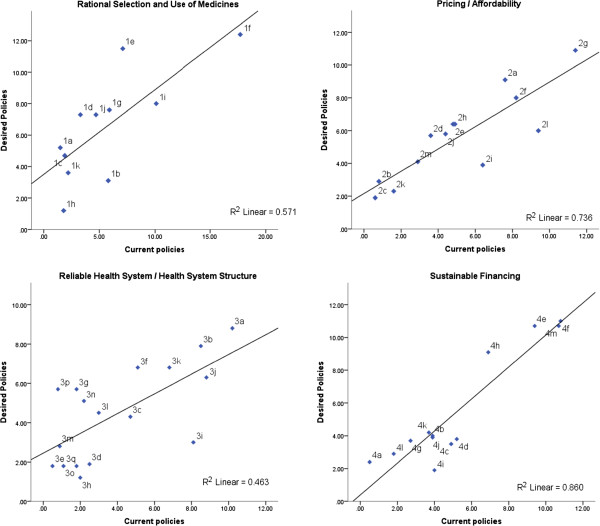
**Scatter plots between summary rankings for current and desired policies, by WHO Framework domain.** Each dot represents a policy/research item.

Analysis of summary rankings indicates a high level of coherence between current and desired policies in all domains: coefficients of determination (R^2^) vary from 0.46 for *Health System Structure* (with a high correlation coefficient; *r* = 0.68, *P* <0.01) to 0.86 for *Sustainable Financing* (*r* = 0.93, *P* <0.01) – current policies, overall, would cover adequate topics (Figure 1).

Despite the general coherence level observed, some items (dots on scatter plots) are distant from trend lines and should receive special consideration. Dots above trend lines represent policies desired by stakeholders but not sufficiently addressed by current policies – representing policy gaps such as item (1e) staff capacity and training for *Rational Selection and Use of Medicines*. The domain with the lowest coherence indicator, *Health System Structure*, presented several gaps: (3p) promotion of research and development – new drugs/neglected diseases; (3g) responsiveness to patients’ needs; (3n) governance: law enforcement; transparency and accountability; and (3f) staff and technical capacity.

#### Is scientific evidence being generated to support policies that respond to the needs of the population?

**Figure 2:**
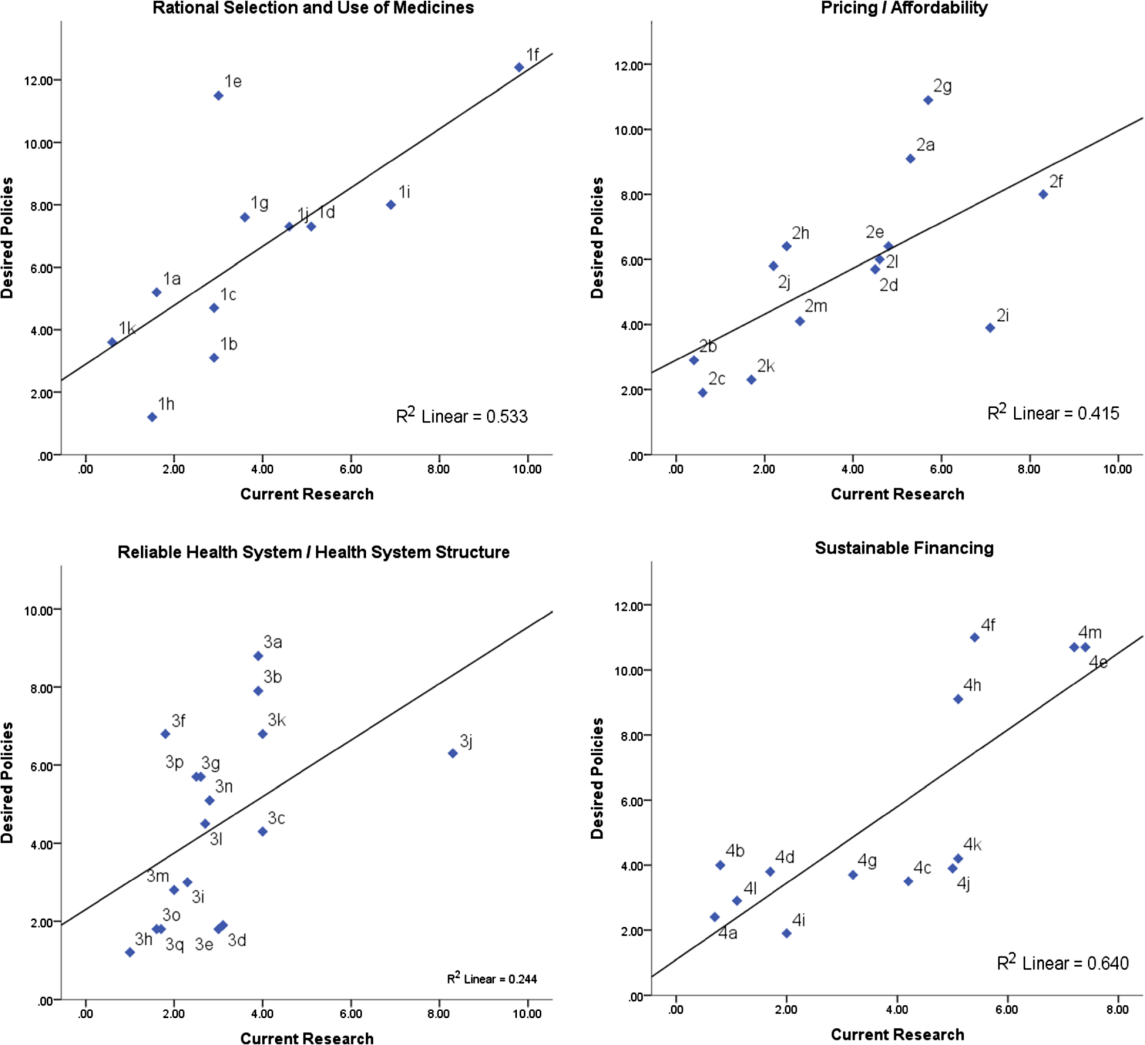
**Scatter plots between summary rankings for current research and desired policies, by WHO Framework domain.** Each dot representing a policy/research item.

Research on *Rational selection and use of medicines* and on *Sustainable Financing* seems to be addressing important policy issues, which is indicated by high coherence between current research and desired policies (*r* = 0.73, *P* <0.05, R^2^ = 0.53; *r* = 0.80, *P* <0.01, R^2^ = 0.65, respectively). However, item (1e), Staff capacity and training for rational prescription and use of medicines (*Rational Selection and Use of Medicines*), is highly prioritized as a desired policy but does not figure amongst the main investigated topics. Concerning *Sustainable Financing*, some items such as (4f) government budgets for medicines and (4h) cost containment policies represent highly desired policies, which are not investigated as much (Figure 2).

The lowest coherence is observed for *Health System Structure* (r = 0.49, *P* <0.05, R^2^ = 0.24). Equally important policy issues as (3f) staff and technical capacity, (3k) quality assurance, and (3j) availability of medicines have different levels of research intensity, with a predominance of the latter (3j). Also, highly prioritized policy issues like (3a) geographical accessibility and (3b) distribution systems and supply chains received much less attention on research. Items (3f), (3a), and (3b) could be considered the most important research topics required to support policies that respond to the needs of the population in this domain.

Coherence between current research and desired policies is moderate for *Pricing and Affordability* (*r* = 0.64, *P* <0.05, R^2^ = 0.41). In this dimension, (2g) policy and regulation for medicines price and (2a) high out-of-pocket payment are high priority issues that should be addressed by public policies but they are not being investigated quite as much. On this domain, research on (2i) patent and intellectual property issues is more intensive, but it does not represent a highly ranked desired policy.

#### Is research moving in the right direction?

**Figure 3:**
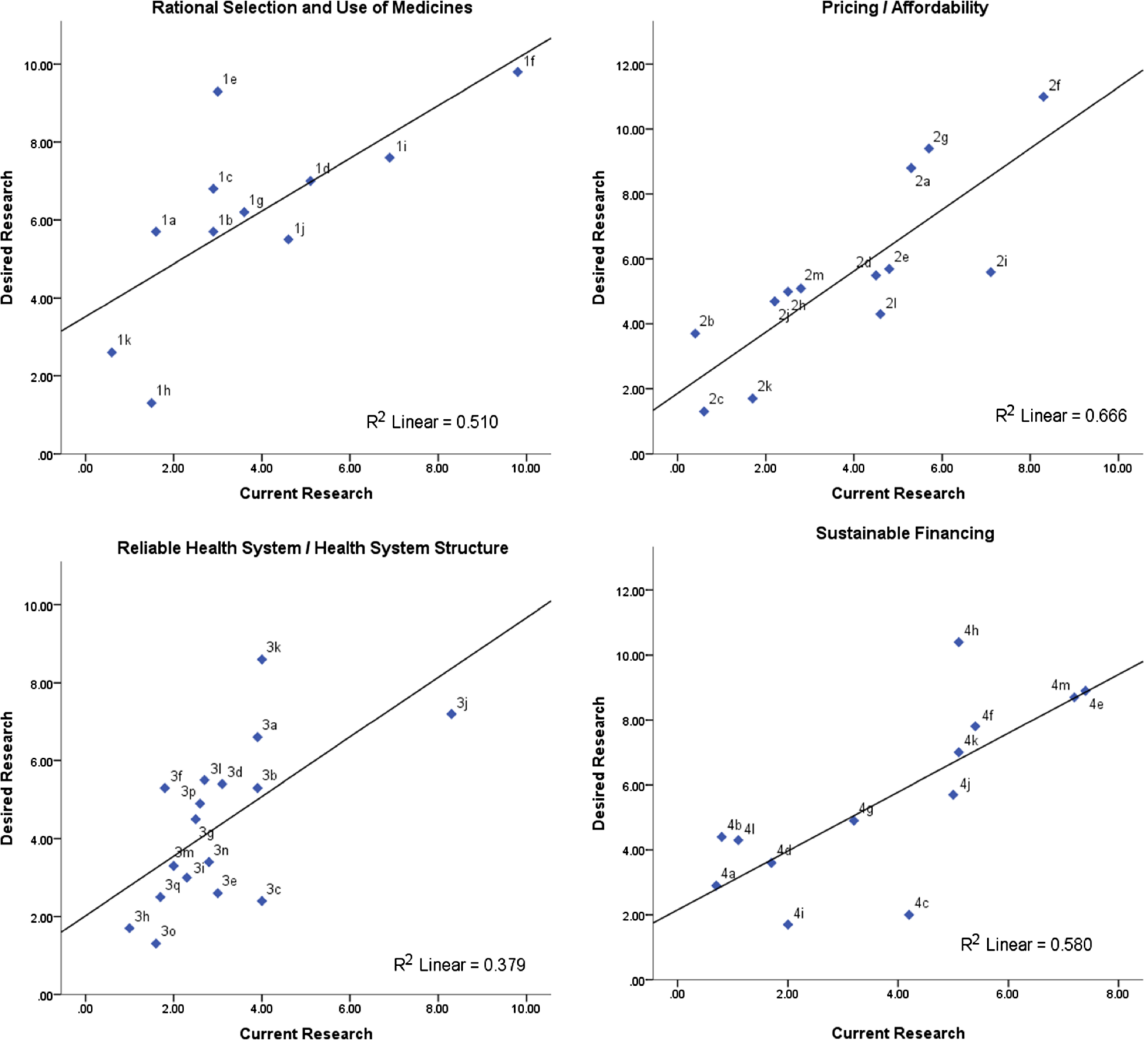
**Scatter plots between summary rankings for current and desired research, by WHO Framework domain.** Each dot representing a policy/research item.

High coherence between current research and desired research indicates that topics currently being investigated at national research centers seem to cover important research topics, required for public health policies on the domains of *Rational selection and use of medicines* (*r* = 0.71, *P* <0.05, R^2^ = 0.51), *Pricing and Affordability* (*r* = 0.82, *P* <0.01, R^2^ = 0.67), and *Sustainable Financing* (*r* = 0.76, *P* <0.01, R^2^ = 0.58) (Figure 3). Staff capacity and training for rational prescription and use of medicines (1e), (2g) policy and regulation for medicines price, (2a) high out-of-pocket payment, and (4h) cost containment policies are highly desired research topics that have not being investigated as much, representing important research gaps on these domains.

*Health System Structure*, the domain with the lowest coherence indicator between current and desired research (*r* = 0.61, *P* <0.01, R^2^ = 0.38), presented several gaps: (3k) quality assurance, (3a) geographical accessibility, and (3f) staff and technical capacity. The latter (3f) presents the same level of research desirability as other items (3l, 3d, and 3b), but received less attention from research initiatives. Distance from trend line makes (3a) the most evident research gap on this domain.

## Discussion

Ranson and Bennett [16] argue that HPSR priorities should emerge from priority setting processes, but adequate processes are rarely performed. They also refer to a WHO survey performed in LMICs showing that often policy-makers, researchers, and users of research are unaware of how priorities are identified or set. Furthermore, priority-setting exercises usually do not reflect a health system perspective and are not “bottom-up”, i.e., does not consider views of stakeholders. This study departed from the organization of issues pertaining to ATM from a health systems perspective [12], and was primarily based upon local level stakeholders’ inputs. Even though policy/research items subject to rankings were developed prior to stakeholders consultation, all ranking lists included an open-ended “other” option with the intention of encouraging respondents to add any issue or topic deemed important and not adequately reflected on the list. That option was rarely used by respondents, which suggests that there was a good level of acceptance and endorsement of the list supplied. Additionally, before being asked to rank policy/research items, participants were asked to expose their concerns on ATM policies and research in an open-ended question, most of the topics or issues that emerged from those answers were similar to items listed in the ranking part of the questionnaire.

According to Viergever et al. [10], there are two main groups of methods that can be applied to set priorities. Consensus-based approaches involve putting people together and in line, allowing participants to reconsider their positions, which tends to improve the acceptability of outputs. However, a consensus-based approach would be difficult for a regional exercise, and has been performed for smaller scale exercises only. Metrics-based approaches, as the one adopted in this study, generate results by pooling individual rankings, which averts the predominance of few most influential participants.

We derived data from individual responses, which were aggregated statistically, aiming at means and averages and looking for communalities rather than disagreements. Therefore, there was no opportunity for participants to confront their views and possibly change their positions to generate consensus. The type of analysis performed is innovative in the field of priority-setting exercises. It establishes major research gaps and policy concerns both directly through the analysis of summary scores, and indirectly through linear regressions. The latter made it possible to identify those policy/research items with a gap between their degree of perceived implementation (current policy/research rankings) and degree of desirability (desired policy/research rankings). This could only be done through the use of a metrics-based approach.

Tromp and Baltussen [17] suggest that an explicit conceptual mapping of criteria facilitates decision making. Even though they discuss priority setting of health interventions, and not health research as focused here, the rationale of aggregating criteria in subsets was useful to reduce the original list of criteria used in our study, and helped define the final list of criteria submitted to respondents. Although we have not used an explicit conceptual mapping of criteria, there was an effort to form groups of criteria with some shared meaning. Instead of predefining which criteria respondents should use when ranking research items, we submitted the prioritization exercise to a previous identification of relevant criteria according to them. This was an attempt to capture respondents’ normative standpoints towards research on ATM. We tried to learn which criteria our pool of interviewees and respondents consider legitimate and relevant and were likely to have influenced their responses. Also, it was a moment to reflect upon those criteria before ranking priorities – which could facilitate decision when answering the questionnaire.

Stakeholder inclusiveness was an important concern of this study, as suggested by Viergever [10]. Even though we cannot assume that participants were statistically representative of all groups involved, we relied on good choice of well-informed respondents and a balance among the type of stakeholders to form an adequate pool of respondents. PAHO local officers’ support was of utmost importance in the identification of relevant national stakeholders for the face-to-face interviews.

Viergever et al. [10] state that priority-setting exercises should be based on agreed criteria. In our study, despite some variation among countries, relevance, feasibility, and research utilization were the most cited. They differ from those agreed in an international consensus exercise on ATM – innovation, impact on health and health systems, equity, and lack of research [13]. This may rely on participants background and context of each exercise. Stakeholders that contributed to our study were asked to base their responses on their countries specificities, while in the international exercise participants were asked to consider the global scenario.

Setting priorities for health policy interventions is not the same as setting priorities for research for health. Groups of stakeholders affected by and interested in policy decisions may differ from those usually interested or involved in research on the same subject. Criteria that are relevant to define policy intervention priorities also differ from those that are adequate to judge how research should be prioritized. This study addressed ATM-related issues, expressed on the set of items submitted to ranking, in which policy interventions can be designed and under which research can be developed. As mentioned above, this regional study contributed to a global initiative to identify research topics under relevant policy issues [13]. Thus, important insight on how to proceed was gathered mainly from literature on research for health priority setting, but also from priority setting on health policy interventions.

Clinical treatment guidelines and essential medicines lists, pertaining to *Rational Selection and Use of Medicines*, received the highest scores, indicating that this is perceived as both an important current policy issue and a highly desired one. It is perceived as a relevant ATM research topic currently being investigated in the region, and also a highly desired research topic. Since 1978, WHO proposes rational selection as one of four key factors influencing ATM, and promotes the adoption of essential medicines lists as a backbone for national pharmaceutical policies [11,18,19]. This aspect was relevant in a broader international research priority exercise [13] and figures among the mostly addressed issues in peer reviewed publications in the region [14].

Staff capacity and training appeared as an important gap, both for policy and for research, and among different ATM domains. The policy gap on staff capacity for rational selection and use of medicines is probably related to the growing need to adopt evidence-based practices [20], which demands skilled staff, generally scarce in LMICs. Due to its importance, the health workforce is stated as one of the health system building blocks [21,22] remaining a worldwide priority and still an important gap [9,23]. A scoping study addressing peer reviewed publication related to ATM in LAC also highlighted human resources and health information as under-represented themes in ATM research [14].

The need for policies addressing the development of new medicines for neglected disease emerged as a gap, showing the lack of treatment options for endemic diseases is still prevalent in the region [24,25]. Tromp and Baltussen [17] recognize responsiveness to the population needs as one of the five categories that reflect the goals of a health system. In our study it emerged as an important policy gap. Another important policy gap, good governance, has been increasingly recognized as necessary for improving access to medicines and contributing to health systems strengthening, since corruption and low transparency is an important cause of wastage of scarce resources [26].

It is remarkable that the same issues appearing as main gaps between current and desired policies – staff capacity and training and cost containment policies – also emerged as important research gaps. Geographical accessibility and distribution systems and supply chains appeared amongst highly desired policy issues that lack research. Geographical accessibility figures as one of the main research gaps. Many countries in Latin America have their population concentrated in big cities, but have large extensions of their territories with low population density, generally low-income regions, not attractive to private medicine suppliers.

This study presents some limitations. The response rate was approximately 36% for the e-mail survey. Nevertheless, this is consistent with the mean response rate (34.6%, SD = 15.7%) reported on a meta-analysis developed by Cook et al. [27] for studies with no missing data. The over-representativeness of academia and NGOs in the e-mail survey was counterbalanced by interviews at country level, where other stakeholder representativeness was greater. A purposive sampling of countries was used and results may not be generalized to other countries.

Different from other prioritization exercises on ATM [13], we have decided – with regional stakeholders – not to detail priorities as specific research questions. Our aim was to map priority topics and to sensitize stakeholders about the importance of evidence-based decision-making and HPSR, motivating them to develop local research priority exercises. Thus, our results should not be taken as top-down advice, but a support to decision makers on the establishment of their own priorities, in which we agree with Tromp and Baltussen [17].

## Conclusions

This study combines strong metrics approaches, such as the coherence analysis, which contributes to priority setting methodology development, with country and regional level stakeholders’ participation. As part of a more comprehensive approach, which included a scoping study of the scientific literature, it achieved useful results both at country and regional levels, and also contributed to a global exercise of research priority settings.

Staff and technical capacity, promotion of Research and Development for new drugs for neglected diseases, and responsiveness to patients’ needs, were the main policy gaps. Human resources technical capacity, medicines price regulation, out-of-pocket payments, cost containment policies, and quality assurance, were the main research gaps identified.

In order to give feedback to the participants and increase the chance of the results being used, an executive summary of the final report was shared with the participants and PAHO officers. Also, results were presented in an internet-based online lecture, with the same audience, apart from having been presented in other technical and scientific meetings. So far, we have not received any feedback on the use of those priorities to support decision-making on visited settings. We believe that the influence of those exercises should merit itself some space in future studies. We hope our study contributes to the discussion and implementation of ATM research and policy priorities in LAC.

## Abbreviations

AHPSR: Alliance for Health Policy and Systems Research; ATM: Access to medicines; HPSR: Health Policy and Systems Research; LAC: Latin America and the Caribbean; LMIC: Low- and middle-income country; MoH: Ministry of Health; NGO: Non-Governmental Organization; PAHO: Pan American Health Organization; WHO: World Health Organization.

## Competing interests

The authors declare that they have no competing interests.

## Authors’ contributions

VLL coordinated the general research of priority mapping in Latin America and the Caribbean, participated in the data collection, and in the conception and writing of this paper. TA acted as adjunct coordinator of the interview component of the general project, participated in the data collection, led the conception of this paper, and actively worked in the text construction. MAO actively participated in the conception of this paper and text construction. IE participated in the general project working team, acting as adjunct coordinator of the first-stage study, participated in the data collection, and collaborated in the text construction. MB coordinated the research priority setting on ATM at global level, giving inputs on methods used in country or regional exercises, contributed to the text construction of this paper. All authors approved the submitted version of this paper.
